# Editorial: Neutrophil extracellular traps: mechanistic and functional insight

**DOI:** 10.3389/fimmu.2024.1407232

**Published:** 2024-04-18

**Authors:** Aneta Manda-Handzlik, Darko Stojkov, Malgorzata Wachowska, Marcin Surmiak

**Affiliations:** ^1^ Department of Laboratory Diagnostics and Clinical Immunology of Developmental Age, Medical University of Warsaw, Warsaw, Poland; ^2^ Institute of Pharmacology, University of Bern, Bern, Switzerland; ^3^ Department of Internal Medicine, Jagiellonian University Medical College, Krakow, Poland; ^4^ Center for the Development of Therapies for Civilization and Age-Related Diseases, Jagiellonian University Medical College, Krakow, Poland

**Keywords:** neutrophil extracellular traps (NETs), inflammation, innate immunity, sepsis, pyroptosis, ANCA-associated vasculitis, rhinitis

Neutrophil extracellular traps (NETs) have emerged as captivating elements within the complex realm of neutrophil biology. Originally renowned for their role in combating infections, neutrophils are now acknowledged for their diverse functions in immune responses and disease processes. NETs, consisting of DNA and proteins (1), effectively immobilize and eliminate pathogens, but their excessive release can trigger detrimental effects to the host (2). Various microbial and noninfectious stimuli have been identified to activate neutrophils and initiate NET formation (3). The production of reactive oxygen species (ROS) plays a crucial role in this process, with glutathionylation of actin and tubulin by the enzyme glutaredoxin 1 (Grx1) being essential for the active cytoskeleton movement required for NET release (4). Metabolic pathways, particularly glycolysis, are vital for the energy demands of neutrophils during NET formation (5). In addition to glycolysis, neutrophils also utilize the pentose-phosphate pathway (PPP) for NADPH and ribose production, and can adapt to fatty acid metabolism and glutaminolysis in certain conditions (6). NETs have been implicated in a pathogenesis of a wide range of diseases, including bacterial infections, autoimmune diseases, thrombosis, cancer, respiratory tract diseases, sepsis, cardiovascular diseases, inflammatory bowel diseases, neurodegenerative diseases, diabetic nephropathy, acute respiratory distress syndrome (ARDS), psoriasis, and COVID-19 (7). Though significant strides have been made in unraveling of NET formation, numerous inquiries and debates remain. Gaining deeper insights into the mechanisms and clinical implications of NET formation holds promise for the development of novel therapeutic interventions.

The present Research Topic primarily discusses the emerging potential therapeutic avenue of targeting NETs in several medical conditions, including sepsis, traumatic brain injury, ANCA-associated vasculitis, and respiratory disorders. It provides insights into the role of Syntaxin-4 and SNAP23 in neutrophil degranulation and NET formation. Additionally, it sheds light on ultraviolet radiation as a trigger to release NETs through DNA nicks, and understanding these mechanisms can lead to the development of new therapeutic strategies.

Early recognition and intervention are crucial in determining the outcome of sepsis, making the clinical assessment of NET release a potentially valuable biomarker for early sepsis diagnosis. Inhibiting the formation of NETs is also considered to be one of the potential treatments for sepsis (8). de Araujo et al. provide new therapeutic approach for sepsis treatment. The Neonatal NET-Inhibitory Factor (nNIF) has a positive impact on survival rates in a model of polymicrobial sepsis known as cecal ligation and puncture. nNIF achieves this by blocking the formation of NETs, which are associated with sepsis. Targeting nNIF can improve survival rates in sepsis by blocking the formation of NETs.

NET markers were previously detected in the plasma of traumatic brain injury (TBI) patients. Coagulopathy contributes to the majority of deaths and disabilities associated with traumatic brain injury (TBI) (9). Previous evidence supports the critical role of NETs contributing to an abnormal coagulation state in the acute phase of TBI. New insights into the role of NET formation in TBI and its impact on neuronal pyroptosis was provided by Cao et al. The study finds that inhibiting NET formation can prevent NLRP1-dependent neuronal pyroptosis in mice with TBI. The study also highlights the involvement of the STING/IRE1α pathway in this process. These findings suggest that targeting NET formation and the STING/IRE1α pathway may present novel therapeutic strategies for mitigating brain damage following TBI.

The generation of ROS by activated neutrophils is one of their fundamental functions. This process can be both positive, facilitating the fight against infection, and negative, leading to tissue damage, e.g. in autoimmune diseases. Neutrophils are known to generate ROS via either NADPH oxidase (NOX) or mitochondria. In a brief research report Azzouz and Palaniyar discussed the process of NETosis, specifically in response to ultraviolet radiation (UV), highlighting the role of mitochondria in generating ROS and how this leads to the formation of DNA nicks. These DNA nicks then trigger the release of NETs. The study provides insights into the molecular mechanisms underlying UV-induced NETosis and suggests that base excision repair steps are involved in this process. Understanding these mechanisms can contribute to a better understanding of the immune response to UV damage and potentially lead to the development of new therapeutic strategies.

The role of mitochondria is not restricted to simply being the source of ROS required for NET formation. Notably, mitochondria can also serve as a source of DNA for the NET scaffold. This aspect of NET release was studied by Gigon et al. The authors focused on the role of two proteins, Syntaxin-4 and SNAP23, in neutrophil’s biology, including degranulation and the release of mitochondrial DNA (mtDNA) during the formation of NETs. The study finds that while these proteins are involved in degranulation and synthesis of ROS, they do not play a role in the release of mtDNA during NET formation. This research provides further insights into various mechanisms and molecules governing neutrophils’ strategies to fight infections, including NET formation.

The antineutrophil cytoplasmic antibody (ANCA)-associated vasculitides (AAV) are a group of rare autoimmune vasculitides characterized by the presence of serum antibodies (ANCAs) directed against neutrophil proteases - proteinase 3 (PR3) or myeloperoxidase (MPO). ANCA-mediated neutrophil activation leads to ROS production, cytokine release, NET formation and subsequent vascular damage. Shiratori-Aso and Nakazawa explored the involvement of NETs in ANCA-associated vasculitis. The article discusses how NETs are released in response to ANCA antibodies, contributing to the pathogenesis of the disease. It also explores the potential role of NETs in promoting tissue damage and autoimmunity in ANCA-associated vasculitis, suggesting targeting NETs as a potential therapeutic approach.

Neutrophils are the most abundant group of white blood cells in adult humans, able to fight infections caused by various pathogens, including bacteria and viruses. However, in protozoan infections such as with Toxoplasma gondii, neutrophils can also contribute to the spread of infection by acting as a reservoir for the multiplication of pathogens. The relationship between human NETs and Toxoplasma gondii infection was discussed by Macedo et al. The authors found that while NETs were formed in response to Toxoplasma gondii, they did not affect the parasite’s ability to infect and replicate within host cells. This suggests that Toxoplasma gondii has developed mechanisms to evade or withstand the effects of NETs, enabling it to establish infection effectively. This study sheds light on the intricate host-parasite interactions and provides insights into the immune responses against Toxoplasma gondii infection.

NETs and their components can be detected not only in infected or inflamed tissues, but also in various biological fluids, including blood, bronchoalveolar lavage fluid (BALF) (10), and cerebrospinal fluid (CSF) (11). Zawrotniak et al. explored the presence and role of NETs in the upper respiratory tract secretions of individuals with infectious and allergic rhinitis. The study found that NETs were present in nasal secretions of individuals with both infectious and allergic rhinitis. Furthermore, the composition and properties of the NETs differed between the two conditions, suggesting that NETs may contribute differently to the pathogenesis of these respiratory disorders. Understanding the role of NETs in rhinitis could provide valuable insights for developing targeted therapies for these conditions.

Overall, the articles compiled in this ‘Research Topic’ offer a comprehensive understanding of the vital role of NET formation. They delve into the mechanisms of NET formation, shedding light on the involvement of specific proteins and mitochondria. These studies also provide valuable insights into the intricate interactions between NETs and infections. By delving further into the formation of NETs and its implications in clinical settings, we can pave the way for novel therapeutic approaches.

## Author contributions

AM-H: Writing – original draft, Writing – review & editing. DS: Writing – original draft, Writing – review & editing. MW: Writing – original draft, Writing – review & editing. MS: Writing – original draft, Writing – review & editing.
